# Exploring the application limits of different hold-up time markers in supercritical fluid chromatography

**DOI:** 10.1007/s00216-024-05152-9

**Published:** 2024-01-25

**Authors:** Csanád Rédei, Alessandro Buratti, Martina Catani, Attila Felinger

**Affiliations:** 1 Department of Analytical and Environmental Chemistry and Szentágothai Research Center, University of Pécs, Ifjúság útja 6, H–7624 Pécs, Hungary; 2 HUN-REN-PTE Molecular Interactions in Separation Science Research Group, Ifjúság útja 6, H–7624 Pécs, Hungary; 3 Department of Chemical, Pharmaceutical and Agricultural Sciences, University of Ferrara, via L. Borsari 46, Ferrara, 44121 Italy; 4 Institute of Bioanalysis, Medical School, University of Pécs, Szigeti út 12, H–7624 Pécs, Hungary

**Keywords:** Supercritical fluid chromatography, Hold-up time, Nitrous oxide, Tracer, Marker

## Abstract

The study focuses on the application range of nitrous oxide as a hold-up time marker in supercritical fluid chromatography (SFC). This compound has been suggested a decade ago to be used as unretained marker, something that the field of SFC was missing for a long time, since its beneficial properties make it an ideal candidate as hold-up time marker. Determination of the hold-up volume and actual volumetric flow rates have always been problematic in SFC due to the compressibility of carbon dioxide and one part of this is the difficulty of hold-up time measurements. Depending on the mobile phase, different methods have been used to measure the hold-up time with varying results. Nitrous oxide and other molecules have been compared in different conditions, mobile phases and stationary phases. In all cases, nitrous oxide gave the lowest elution times. However, detection was difficult in mobile phases containing 10% or more of organic modifier, because most solvents mask the signal of nitrous oxide. Interestingly, the choice of stationary phase also had a slight effect on detection, while different pressure and temperature settings affected each compound in a different manner.

## Introduction

The compressibility of carbon dioxide results in the variation of a series of chromatographic properties in supercritical fluid chromatography (SFC), since apart from the pressure drop along the column, the mobile phase also undergoes significant expansion. Density is the most crucial property, but viscosity, thermal properties and solvation strength of the eluent are also affected, in addition to retention factors, column efficiencies, diffusivities and solubilities of the sample components. Due to the nature of carbon dioxide, selecting the working point around the critical region of the mobile phase is a complicated task. However, it can be aided by the use of isopycnic lines in order to better understand the behavior of the physical properties of carbon dioxide as shown by Tarafder et al. [1–4]. Method transfer and scalability introduce additional challenges to SFC practitioners; however, more recent solutions provide new tools to overcome the obstacles [5].

It is also understood that in SFC the set and true volumetric flow rates differ from each other, which can become an important issue when trying to translate retention times and hold-up times into retention volumes and hold-up volumes, respectively. Similarly, the method of hold-up time measurements is not as universal as in liquid chromatography. Previously, system peaks, solvent peaks, minor disturbance peaks or compounds considered unretained have been used for hold-up time measurements, while in chiral SFC acetone or 1,3,5-tri-*tert*-butylbenzene (TTBB) have been employed [6, 7]. However, Trebel et al. proved through molecular simulations that acetone demonstrates similar behavior to the co-solvent acetonitrile and the analyte acetophenone in reversed phase liquid chromatography (RPLC) [8]. Regarding TTBB, West et al. pointed out that the use of this compound as hold-up time marker is common in normal phase LC, but shows significant retention on non-polar stationary phases [9].

The use of nitrous oxide (N_2_O) as a hold-up time marker for supercritical fluid chromatography has been established for a decade now with the first applications published by Vajda et al. in 2013 [10–12]. Nitrous oxide displays beneficial properties that make it an ideal hold-up time marker such as low to negligible adsorption on the stationary phase, low dipole moment, high vapor pressure and low concentration required that prevent any competition with other mobile phase components for the adsorption sites while also providing well-resolved peaks. Sample preparation is easy due to the relatively good solubility of nitrous oxide in alcohols.

The goal of this paper is to further explore the applicability of nitrous oxide as a hold-up time marker in SFC using a wider range of experimental conditions regarding column temperature and back pressure while also studying different stationary phases, mobile phase compositions and making comparisons with other molecules used as hold-up time markers.

## Materials and methods

### Chemicals and columns

Carbon dioxide ($\ge$99.5%) was obtained from Linde (Répcelak, Hungary) while HPLC grade methanol ($\ge$99.9%) and hexane ($\ge$95%) were obtained from Fisher Scientific (Loughborough, UK). Nitrous oxide was purchased from Messer (Lenzburg, Switzerland). 1,3,5-tri-*tert*-butylbenzene (97+%) was purchased from ThermoFisher GmbH (Kandel, Germany). Trifluoroacetic acid (TFA, $\ge$99.0%) was purchased from Sigma-Aldrich Chemie GmbH (Steinheim, Germany). Acetone (distilled) was provided in-house by the Department of General and Inorganic Chemistry (University of Pécs, Hungary)

The columns employed in the study were two Spherisorb Silica columns (5 and 10 $\upmu$m, $4.6\times 100$ mm), a Symmetry C_18_ (3 $\upmu$m, $4.6\times 150$ mm), a Viridis BEH (1.7 $\upmu$m, $3.0\times 50$ mm) and a Torus Diol (1.7 $\upmu$m, $3.0\times 50$ mm), all from Waters (Milford, MA, USA) along with a Supelcosil ABZ+Plus alkylamide column (3 $\upmu$m, $4.6\times 150$ mm) from Sigma-Aldrich (St. Louis, MO, USA) and a (S,S) Whelk-O1 (4.6 $\times$ 100 mm), packed with fully porous, 1.8 $\upmu$m particles synthesized at the Department of Chemical, Pharmaceutical and Agricultural Sciences (University of Ferrara, Italy) [13].

### Instruments

The experiments were performed utilizing two SFC systems. The first one was a Waters ACQUITY UPC^2^ system equipped with a binary solvent delivery pump, an autosampler fitted with a 10 $\upmu$L sample loop, a column thermostat, a PDA detector and a back pressure regulator (BPR). The instrument was controlled by Empower 3 chromatography data software.

The second system was an Agilent 1260 Infinity II SFC System equipped with a binary pump, multisampler, multicolumn thermostat, diode array detector and SFC control module. The instrument was controlled by Agilent ChemStation software.

Mass flow rate of the mobile phase was measured with a mini CORI-FLOW mass flow meter from Bronkhorst High-Tech B.V. (Ruurlo, Netherlands), Model No. M13-ABD-11-0-S, Serial No. B11200776A. This model provides an accuracy of ±(0.2% of the read value + 0.5 g/h), expressed as a sensitivity of 0.01 g/min of CO_2_.

### Experiments

All experiments were performed with a set flow rate of 1.00 mL/min. The mobile phase is specified in each subsection. The injection volume was 2.0 $\upmu$L. The detector signal was recorded between 190 and 400 nm. Four different sets of settings were used for the column thermostat and back pressure regulator as shown in Table 1.

**Table 1:** The four sets of settings for the column thermostat and back pressure regulator

	T (^∘^C)	p (bar)
A	20	104
B	20	150
C	40	104
D	40	150

**Fig. 1:**
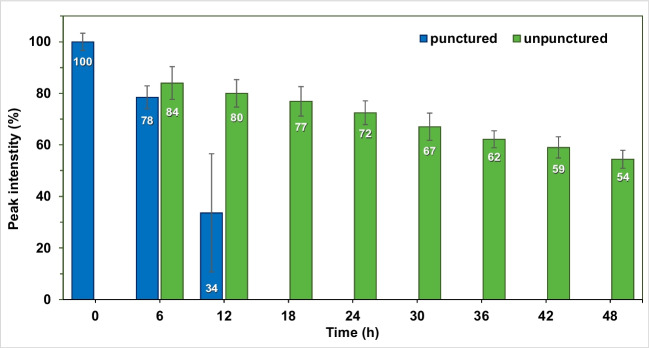
Stability of the nitrous oxide sample over a period of 48 h

Stability of the nitrous oxide sample, accounting for punctured and unpunctured vials, was measured using the alkylamide column, 100% CO_2_ mobile phase, 20 ^∘^C column temperature and 104 bar back pressure. Stock solution was prepared with 1 min bubbling in methanol, so that all vials contained the same initial concentration of nitrous oxide. The time period covered was 48 h in 6 h increments. 9 vials were prepared, each containing 1 mL stock solution. The first vial provided the 100% peak intensity (0 h) and would remain punctured throughout the 48 h. The other 8 vials accounted for the injections at 6, 12, 18, 24, 30, 36, 40 and 48 h. During the sequence, injections were made from the first vial and then also at each increment, while the vial stayed punctured. Starting at 6 h, injections were made from an unpunctured vial too, and then at each increment a new vial was punctured. Three replicate measurements were performed for each vial. The autosampler was thermostated at 25 ^∘^C.

## Results and discussion

The first part of the section focuses on sample preparation and stability studies of nitrous oxide; then, some early studies of possible tracer compounds are discussed (“Preliminarystudies” section). Next, a more detailed comparison is made between nitrous oxide and other markers that have been used in different fields of SFC (“Comparison of different markers” section). After that, the effect of the organic modifier, namely methanol is studied in detail (“The effect of the organicmodifier” section). Then, we look into the possible effect of the stationary phase (“The effect of the stationary phase” section). It is important to note, that only results related to 20 ^∘^C column temperature and 104 bar back pressure (condition A in Table 1) are presented and discussed in these sections. However, the final section addresses and evaluates the effect of different experimental conditions (pressure and temperature) on the elution of the tracer compounds (“The effect ofpressure and temperature” section).

### Preliminary studies

Our early studies focused on method development for proper nitrous oxide detection. For this purpose, several experiments were performed with neat methanol, hexane and nitrous oxide dissolved in methanol, all three used as possible unretained markers. The chosen default conditions were neat carbon dioxide mobile phase, 20^∘^C and 104 bar back pressure with the alkylamide column. The experiments were performed with and without the Coriolis flow meter connected to the system.

The results showed that nitrous oxide was the best compound out of the three since it exhibited the lowest elution time. Due to the neat carbon dioxide mobile phase, methanol was immediately adsorbed on the stationary phase and was eluted with a large diffuse band at later retention times. Hexane eluted slightly later than nitrous oxide; however, its signal was unsuitable for evaluation due to disturbances detected throughout the entire wavelength range. Experiments performed with and without the CFM connected upstream and downstream the column proved that the unit should not be connected for separations due to the large volume and band broadening effect added [14]. In addition, the CFM also induced very slow equilibration of the pressure and sometimes even a slight malfunction of the back pressure regulator. This error was also present when the CFM was connected to positions other than the vicinity of the column.

Sample preparation time of nitrous oxide was investigated as well, with 1, 2 and 5 min of bubbling of the gas in methanol. Injections made with the three samples showed that time had no significant effect; intensities, peak areas and peak widths were practically the same in each case, meaning that nitrous oxide probably reaches its maximum concentration in methanol in under a minute. Based on these findings, 1 min should be sufficient for sample preparation.

**Fig. 2:**
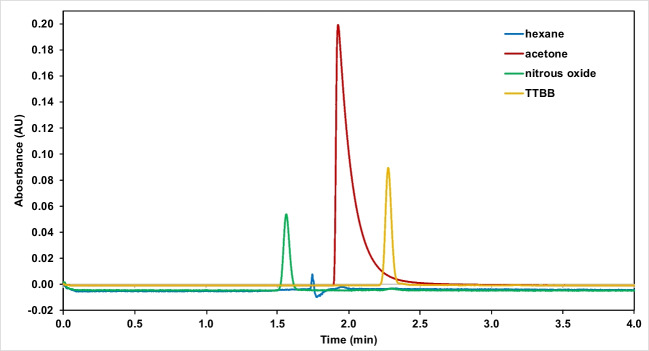
Comparison of different hold-up time markers in the case of the alkylamide column, 20 ^∘^C and 104 bar

Stability of the nitrous oxide sample was also studied. Literature states that the sample in unstable and a fresh one should be prepared every couple of hours [11]. The results are summarized with RSD% values in Fig. 1. The results show that by 18 h, nitrous oxide completely evaporated from the punctured vial and could not be detected anymore. In fact, the high RSD% (23%) observed at 12 h is the result of the third replicate showing a 13% decrease in peak intensity compared to previous replicates for this vial. Generally, the RSD% was above 1% in each case and varied between 3 and 6%. This is due to each measurement running for 20 min in order to completely elute methanol from the column before the next injection, which leaves enough time for the concentration of nitrous oxide to decrease, even in the unpunctured vials. The unpunctured vials also showed that nitrous oxide was retained in the sample even for 48 h.

### Comparison of different markers

After the preliminary studies, different molecules were compared that have been used previously as hold-up time markers in SFC, namely hexane, acetone (10 V/V% in MeOH), nitrous oxide (1 min in MeOH) and TTBB (0.01 mg/mL). Neat acetone proved to be too concentrated resulting in distorted peak shapes hence the dilution in methanol. A series of experiments were performed using two of the columns, Spherisorb 5 $\upmu$m and ABZ+Plus alkylamide, and the four sets of settings for the column thermostat and back pressure regulator detailed in Table 1 (Settings A through D). The mobile phase was neat carbon dioxide.

The results showed that hexane behaved in each case as expected; unreliable, noisy signal with disturbances and impurities. In the case of the silica column, the elution time was equal to that of the nitrous oxide signal, while for the alkylamide column a slight retention could be observed. Nitrous oxide exhibited optimal, well-resolved peaks for all experiments and again proved to be the best marker for hold-up time. Acetone was significantly retained in all cases. Moreover, it also overloaded both columns, detected as a large solvent band with a diffuse rear part followed immediately by the similar solvent band of methanol. TTBB was also retained in all conditions, although to a lesser extent, detected around 0.2–1 min later than nitrous oxide. Figure 2 shows the comparison of the four markers in the case of the alkylamide column, 20^∘^C and 104 bar (Setting A). For the sake of proportionate comparison, hexane and N_2_O were plotted at 195 nm, while acetone and TTBB were plotted at 270 nm.

**Fig. 3:**
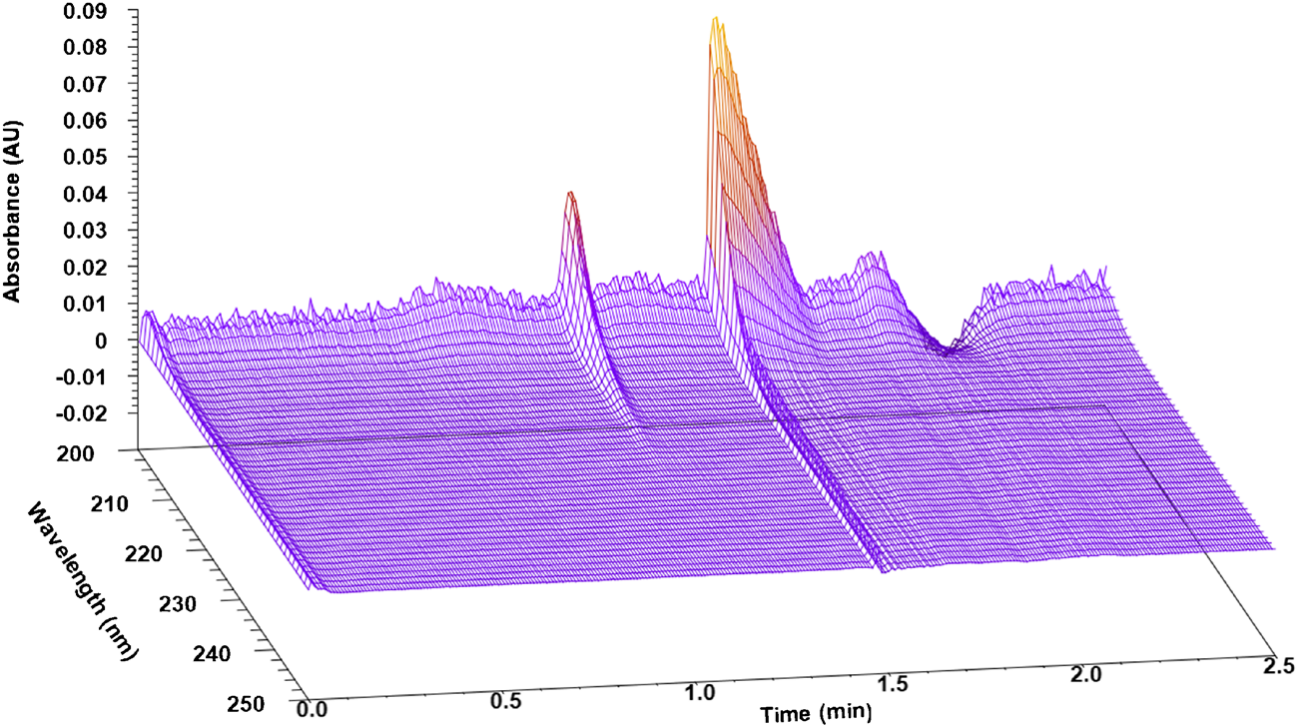
PDA spectum of nitrous oxide at 5% of methanol on the Spherisorb 5 column

**Fig. 4:**
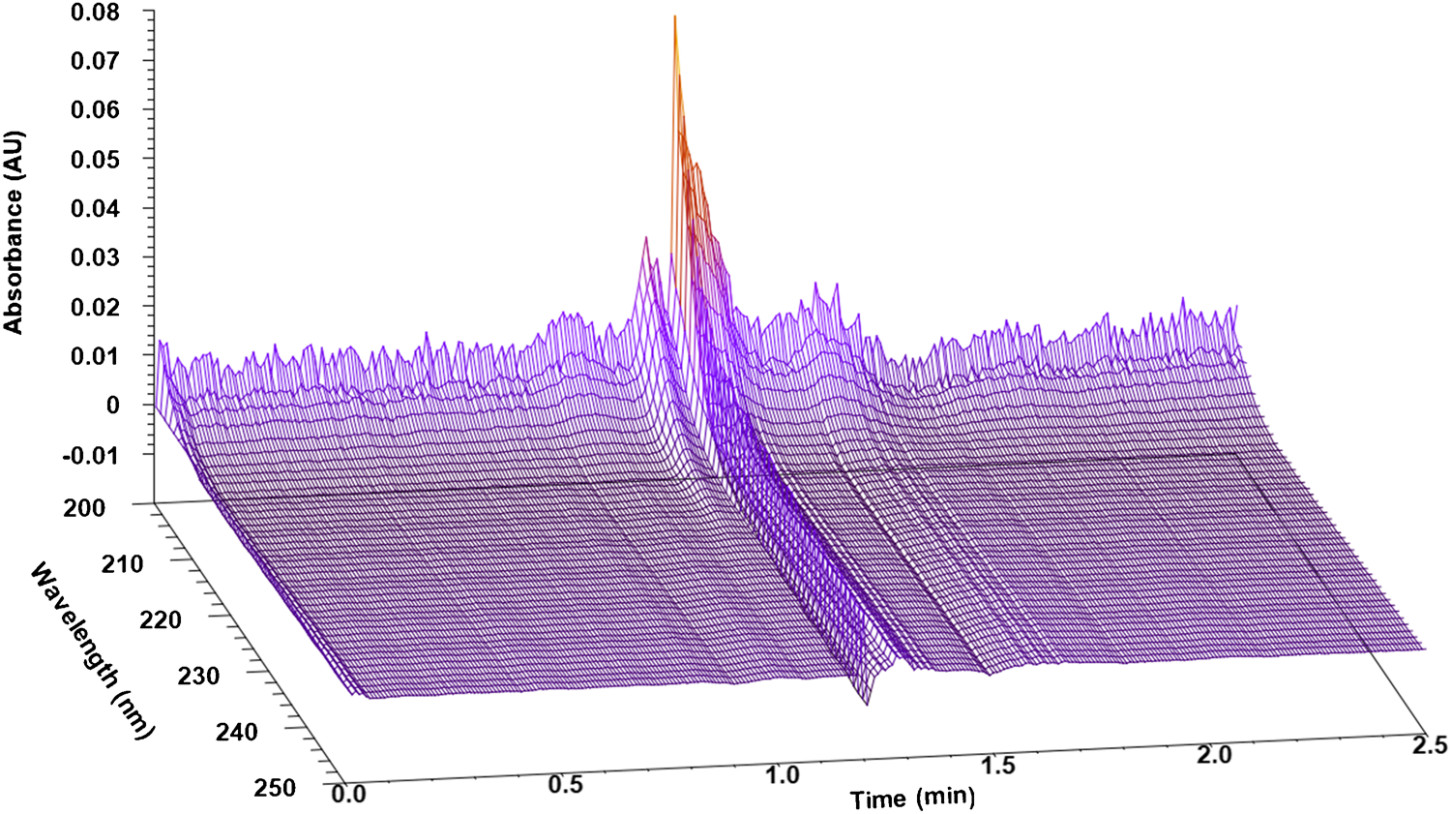
PDA spectum of nitrous oxide at 10% of methanol on the Spherisorb 5 column

### The effect of the organic modifier

The next factor investigated was the amount of organic modifier in the mobile phase. For this purpose, a series of measurements were carried out with mobile phases containing 5, 10, 15 and 20% of methanol as organic modifier. The literature provides accurate methods aimed to determine the exact mass or volume fraction of the organic modifier in the mobile phase, as shown by the group of Fornstedt [5]. This time, the exact composition had no important role in, e.g., numerical calculations, therefore, only the nominal composition provided by the instrument pumps was considered for the sake of observation and comparison. The samples, temperature and back pressure settings were the same as in “Comparisonof different markers” section. The mobile phase compositions were tested on the Spherisorb 5 column, while for the alkylamide column only 5% of methanol was used.

In the case of the silica column and at 5% of methanol, nitrous oxide could be detected with a reliable peak shape and good S/N ratio, although with very little intensity in the range of 200–220 nm. The chromatogram of hexane was again scattered with disturbances and noise; however, the general elution time was very similar to the elution time of nitrous oxide with methanol present in the system. Over 210 nm, hexane gave a signal closely resembling system peaks in LC. Acetone and TTBB were still retained, although their detection was much easier due to their distinct local absorbance maxima at around 270 nm.

One of the most important hardships of N_2_O measurements in SFC is the UV cutoff wavelength of most organic solvents. Unfortunately, this was the case at 10, 15 and 20% of methanol in the mobile phase, since this solvent has a cutoff wavelength of 205–210 nm and thus masked the signal of nitrous oxide, whose concentration in the sample was already very low. Acetone and TTBB could be detected, but with significant fronting. For comparison, Fig. 3 shows a PDA spectrum of nitrous oxide recorded at 5% of methanol, while Fig. 4 shows the spectrum recorded at 10%. The former shows how nitrous oxide could easily be detected at around 1.1 min using 5%, while the latter shows that at 10% the signal was almost nonexistent and merged into the distorted system peak caused by the sample solvent.

In the case of the alkylamide column and 5% of methanol, none of the markers provided usable signals. Hexane showed similar behavior as before, acetone and TTBB gave signals with shouldering and significant fronting, and nitrous oxide could not be detected at all, so this column was not pursued any longer.

Additional testing was performed with the (S,S) Whelk–O1 chiral stationary phase using 0–5% of methanol and our findings were very similar to those observed previously. Acetone gave a signal closer to the estimated hold-up time, although shouldering was also observed. Nitrous oxide could not be detected if any amount of methanol was present in the mobile phase. We also tried adding 0.1% of TFA to the organic modifier in the hopes of covering some of the adsorption sites of the stationary phase, but unfortunately methanol still masked the signal of nitrous oxide.

### The effect of the stationary phase

In theory, the stationary phase should not affect the behavior of unretained markers, especially nitrous oxide that should not exhibit any adsorption or competition on most adsorbent surfaces. In any case, a selection of stationary phases (“Chemicals and columns” section) were tested with the exception of the previously studied columns (Spherisorb 5, ABZ+Plus and (S,S) Whelk–O1). The samples were once again the same as detailed in “Comparison of differentmarkers” section. The mobile phase contained 5% of methanol as organic modifier.

**Fig. 5:**
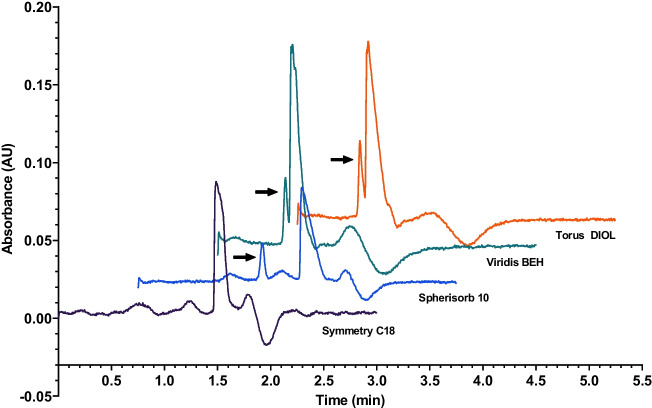
Detection of nitrous oxide on various columns at 5% of methanol and 200 nm

**Fig. 6:**
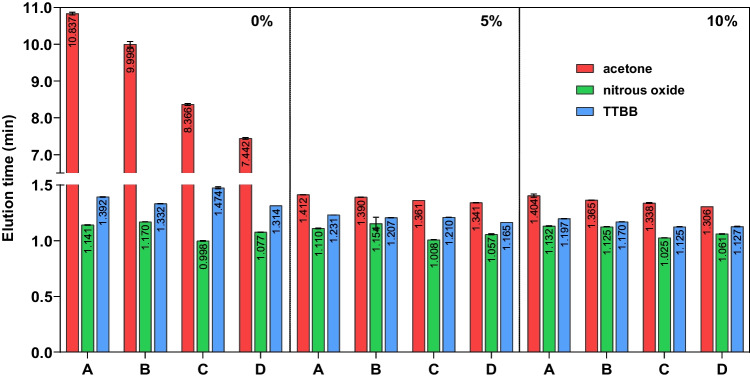
The effect of pressure and temperature, as well as modifier content in the case of the Spherisorb 5 column. A, B, C and D denote the different settings of Table 1, while the organic modifier content is marked in the upper right corner of each subplot

On the Symmetry C_18_, acetone was slightly retained and gave a signal just after the system peak. TTBB had significant retention, as anticipated, and nitrous oxide could not be detected unfortunately. Spherisorb 10 behaved very similarly to Spherisorb 5 since the only difference in the columns was the particle size. Nitrous oxide could be detected, although barely, while acetone and TTBB were retained.

Nitrous oxide was a suitable marker for Viridis BEH and Torus DIOL, while the other samples gave unreliable and unclear signals. Figure 5 shows a comparison of nitrous oxide chromatograms on the different stationary phases. The black arrows mark the peaks of nitrous oxide in each case. The different elution times (1.169 min for Spherisorb 10, 0.638 min for Viridis BEH and 0.590 min for Torus DIOL) are due to the different column dimensions as well as pore size distribution, packing quality and nature of the stationary phases. Due to the compressible mobile phase, the hold-up time is related only to an apparent dead volume, also depending on the varying degree of adsorption of the mobile phase components on the stationary phase.

### The effect of pressure and temperature

To study the effect of column temperature and back pressure, four different set of settings were employed (Table 1). The first one, setting A, aimed to provide subcritical conditions with 20 ^∘^C temperature and 104 bar back pressure. Even though 104 bar is above the critical pressure of neat carbon dioxide, this was the lowest the instrument allowed, and so was considered a sort of low-pressure subcritical setting. Setting B was aimed as a high-pressure setting with 20 ^∘^C and 150 bar. Setting C was a high-temperature setting with 40 ^∘^C and 104 bar. For neat CO_2_, these settings would already provide a low-pressure supercritical state. Setting D was aimed to provide a high-temperature and high-pressure environment with 40 °C and 150 bar. All experiments detailed in the previous sections were performed with all four column temperature and back pressure settings.

The results showed varied trends in the elution behavior of the investigated markers. Hexane was omitted from the evaluation due to inconclusive peak shapes. Generally, temperature and pressure had varied, but mostly slight effects on the elution of the different compounds. The observations are best summarized in the case of the Spherisorb 5 column due to the highest range of mobile phase organic modifier content covered here, while on the other columns, only 0 or 5% (V/V) of methanol was investigated. Figure 6 shows how in the case of neat CO_2_, the retention time of acetone (which overloaded the column) gradually decreased, regardless of which parameter was changed. Retention time was determined at the point where the diffuse rear part of the peak returned to the baseline; however, the same trend was observed for the peak maximum. A gap was inserted in the *y* axis in order to better show the rest of the results. While not overloading the column anymore at 5 and 10% methanol content, acetone continued the same decreasing trend for the rest of the experiments.

Nitrous oxide exhibited behavior contrary to that one would expect in SFC. Increasing the back pressure to 150 bar (setting B) also slightly increased the elution time, despite the density, viscosity and diffusivity also increasing with increased pressure. Retained compounds, which also interact with the stationary phase, normally exhibit a decrease in retention time, such as the weakly retained TTBB in our case. Increasing the temperature only, from 20 to 40 ^∘^C (setting C), decreased the elution time of nitrous oxide, despite the decreased density and viscosity of the mobile phase. TTBB showed increased retention time compared to setting A. Increasing both the back pressure and temperature together (setting D) set the elution time of nitrous oxide between those observed for settings B and C, while TTBB exhibited the lowest retention time out of the four pressure-temperature settings, even compared to setting A. These trends continued also when the mobile phase contained 5 or 10% organic modifier.

## Conclusions

Nitrous oxide can be an ideal hold-up time marker in supercritical fluid chromatography. It has good solubility in alcohols, sample preparation is simple and the sample is stable enough for a day. In addition, the properties of the gas guarantee that no adsorption or competition should take place on the stationary phase during an SFC run. The preliminary studies showed that out of all tested markers, nitrous oxide demonstrated the lowest elution times, suggesting that it was unretained, while the other probes showed some amount of retention or distorted, noisy peak shapes.

However, detection was problematic, because the signal of nitrous oxide had very low intensity due to the low concentration in the sample. Its UV spectrum is also unfortunate, because the molecule can be detected only in the range of 190–220 nm, where most of the organic modifiers employed in SFC have their cutoff wavelengths. Thus, the signal is masked as seen on many occasions during our experiments, especially if the mobile phase contains 10% of methanol or more.

The comparison of different columns implied that the stationary phase did have a slight effect on the detection of nitrous oxide, however, this would contradict the theory behind the use of the molecule as unretained marker and thus must be a result of the structure of the stationary phase only and not the chemistry. Interestingly, the comparison of different pressure and temperature settings showed different trends in behavior for each compound. With an even more comprehensive study, the limit of application could be further extended.
